# Clinical severity of RSV bronchiolitis

**DOI:** 10.1002/hsr2.543

**Published:** 2022-03-22

**Authors:** Faris Hussain, Margarita Delgado Thompson, David Vick, Jack West, Martin Edwards

**Affiliations:** ^1^ Cardiff and Vale University Health Board, NHS Wales University Hospital of Wales Cardiff UK; ^2^ Children's Hospital for Wales, Cardiff and Vale University Health Board, NHS Wales University Hospital of Wales Cardiff UK

**Keywords:** epidemiology, infectious diseases, paediatrics, public health

## Abstract

**Background:**

Studies comparing the severity of respiratory syncytial virus (RSV) bronchiolitis to other viruses are inconclusive. Our study aimed to compare the severity of bronchiolitis according to the virus.

**Methods:**

Data were collected from 1152 infants under one year of age admitted to Children's hospital for Wales, over the winter months of 2014–2020. The cohort was divided based on the virus detected: RSV, RSV with another virus, or other virus. Using *t* tests and Fisher exact statistical test, the groups were compared based on length of hospital stay, admissions PICU/HDU, intubations, and need of nasogastric (NG) nutritional support.

**Results:**

Fifty‐six percent throat swabs were RSV positive, 15% had RSV with another virus, and 29% had only another virus. Children positive for RSV had statistically longer hospital admissions and were more likely to need NG nutrition; however, there was no difference between number of PICU/HDU admissions or intubations. The RSV group and RSV with another virus group had no statistical differences.

1

1.1

Bronchiolitis is a leading cause for hospital admissions in infants. It is caused by a number of respiratory viruses; the most common being respiratory syncytial virus (RSV). Multiple studies demonstrate the overall burden of cost to the health service of RSV, supporting the need for prevention and improved clinical management.^1^ However, there are fewer studies comparing the burden of RSV bronchiolitis to that of other viruses causing bronchiolitis.^2^ With the advent of new vaccines and the emergence of a bronchiolitis epidemic post‐COVID‐19 restrictions being lifted,^3^ more evidence is needed comparing healthcare resource consumption of patients with RSV in comparison to those infected by other viruses. The aim of our study was to identify whether children with RSV bronchiolitis required higher levels of care than children infected with non‐RSV bronchiolitis.

Data were collected retrospectively from children admitted to the Children's Hospital for Wales (CHfW) over the winter months of 2014–2020. Further details of data collection can be seen in our previous report.^3^ The study included infants admitted to the hospital who were coded with bronchiolitis under the International Classification of Diseases Tenth Revision codes (J10‐18, J20‐22, J45, and J46). The exclusion criteria included patients discharged directly from accident and emergency or the assessment unit, children with extreme prematurity (under 32 weeks gestation), and children with congenital heart disease, lung disease or neurological disability, as these patients could have been eligible for RSV prophylaxis as outlined by the NICE guidelines.

As all data were retrospective and anonymised, no consent was needed from the families. Results were manually inputted into excel and statistical comparisons were made between the groups using Fisher exact analysis and *t* tests through STATA SE 16.

We compared the viral results on oropharyngeal swab to the treatment required by the child. The cohort was divided based on the results of the swabs into patients with RSV alone, patients with RSV and another virus, and patients with another virus. The outcomes used were: length of hospital stay, need for paediatric intensive care unit or high dependency unit care (PICU/HDU), and requirement for intubation and requirement for nasogastric nutritional support (NG). These outcome measures were chosen because of the reliability of data available and the reflection of both respiratory and nutritional support. The need for NG nutritional support could not be collected before 2016/2017 as the electronic discharge summaries were not available.

Our results showed over the six winters a total of 3839 patients presented with bronchiolitis to the Children's Hospital for Wales. A total of 1517 infants were admitted to the hospital with bronchiolitis. Of these patients, 415 patients were excluded from the study based on the exclusion criteria, or due to patients not having oropharyngeal swabs completed. From the cohort of patients in the study, 1102 oropharyngeal swabs were collected and 976 tested positive for a virus.

The baseline characteristics of the patients are presented in Table 1. Of the patients with positive swabs, 591 were male (60.6%) and 385 (39.4%) were female. Of the patients admitted to the ward, 107 infants were premature (11.0%); any infants born with a gestation age less than 32 weeks or with significant co‐morbidities were excluded. The percentage of premature patients varied between 9.0% in the RSV group and 14.1% in the group positive for a different virus. There were statistical differences between these two groups (*p* < 0.05).

**Table 1:** Demographics

	RSV alone	RSV with another virus	Other virus alone	Total
Total patients admitted	546	146	284	976
Gender				
Male	307	102	182	591
Female	239	44	102	385
Average age on admission				
Days	113.8	136.0	169.7	133.4
Number of Preterm (32–37 weeks)	49 (9.0%)	18 (12.3%)	40 (14.1%)	107
Socioeconomic				
WIMD*	824.4	772.2	852.8	824.7
WIMD for PICU patients	783.5	778.7	884.3	854.6

Abbreviations: RSV, respiratory syncytial virus, WIMD, welsh index of multiple deprivation.

In total, 55.9% of patients tested positive for RSV alone, 15.0% tested positive RSV in combination with another virus and 29.1% tested positive for a virus or viruses other than RSV.

The patients presenting with RSV alone had an average length of admission of 3.76 days, compared with 4.18 days if another virus was present (Table 2). The patients negative for RSV had an average stay of 3.20 days. This was statistically shorter than the RSV positive patients (*p* < 0.05). There was however no statistical difference between the two groups positive for RSV (*p* > 0.05).

**Table 2:** Summary of outcomes based in the virus category of the patient

Virus category	RSV alone	RVS with another virus	Other virus alone	Total
Total number of patients	**546**	**146**	**284**	**976**
Average length of admission (Days)^a^	3.76	4.18	3.20	3.66
Number of PICU/HDU admissions	69	17	30	116
Average length of PICU stay	3.90	4.00	2.99	3.68
Number of patients requiring intubation	16	4	8	28
Total number of patients with nutrition recorded^b^	**367**	**112**	**216**	**695**
Number of patients requiring NG nutrition^a^	276	83	123	482

^a^ Statistically significant to a *p* < 0.05.

^b^ Data collected from after 2015/2016 due to lack of data before this.

The percentage of admissions to PICU/HDU in the RSV alone group was 12.6%, compared with 11.6% in the RSV and another virus group and 10.6% in the RSV negative group. The length of PICU/HDU stay varied between 3.90 days in the RSV alone group, 4.00 days in the RSV and another virus group and 2.99 days in the RSV negative group. From the RSV alone group 2.9% required intubation compared with 2.7% when RSV was present with another virus and 2.8% when RSV was negative. There was no statistical difference seen in percentage of PICU/HDU admission, length of PICU/HDU stay or percentage of patients requiring intubation (*p* > 0.05). Finally, in the RSV alone group 75.2% required NG nutrition compared with 74.1% of those with RSV and another virus and 56.9% of those with another virus alone. There was a statistical difference between the groups with RSV compared with the RSV negative group (*p* < 0.05), but no difference between the two RSV positive groups (*p* > 0.05).

The aim of this study was to add to the body of evidence about the viruses that cause bronchiolitis. The results show that the majority of our patients tested positive for RSV. The patients positive for RSV were more likely to need longer hospital admission and were more likely to require NG nutritional support. However, there were no statistical differences between number or length of PICU admissions and number of patients requiring intubation.

One strength of our study is that it included data from the largest children's hospital in Wales, and is the first study from this region. One limitation of this study is we were unable to pair the infants for age, gender and gestation, which could limit external validity of our results.

We found that the RSV group was statistically younger, more likely to be female, and had a lower percentage of preterm infants. This may have contributed to the outcomes we observed, as younger infants are known to have more severe bronchiolitis.^4^ However, being female and born at term are protective factors in bronchiolitis.^5^, ^6^

The only prevention for bronchiolitis is pavulizumab, a monoclonal antibody for the RSV virus.^7^ The medication is prohibitively expensive, depending on birth weight and time of year the baby is born the protection for an entire RSV season is between $3221 and $12,568 USD.^7^ A cost analysis study to assess whether expanding the remit for RSV vaccination would be of benefit to patients. Given the emergence of RSV bronchiolitis cases in the UK out of season, including in our own hospital,^3^ consideration should be had as to whether preventative treatment for RSV bronchiolitis should be rolled out to a greater at risk population. Recent analysis of 2020/2021 season data showed that RSV cases dropped significantly during this season; however, following the lifting of COVID‐19 restrictions in the UK, RSV cases have risen dramatically in many westernised countries, including Wales.^3^

In conclusion, this study supports the hypothesis that RSV causes a more severe bronchiolitis than other viruses given the increased hospital stay and need for nutritional support.

## PATIENT AND PUBLIC INVOLVEMENT

Patients were not directly involved in this retrospective data analysis.

We developed our analysis of the retrospective data by considering what is already known about RSV bronchiolitis and how this analysis could expand that knowledge. This would be helpful for considering managing future patients with bronchiolitis.

Patients or the public were not involved in the design or retrospective analysis if the data, as the patient data was anonymised. It was not appropriate or possible to involve patients or the public in the design, or conduct, or reporting, or dissemination of plans of our research

Patients or the public were not involved in the recruitment to and conduct of the study. The data was anonymised and received through the University health board audit department.

## CONFLICTS OF INTEREST

MOE declares payment from Abbvie to attend an RSV discussion panel. Abbvie were not involved in the study design; collection, analysis, and interpretation of data; writing of the report; the decision to submit the report for publication.

## ETHICS STATEMENT

No formal ethics committee carried out approval of this retrospective analysis. This analysis was conducted in accordance with the recognized standards of the 2013 updated Declaration of Helsinki. No patient identifiable information was collected as part of the analysis of this study. It was deemed not appropriate or possible to involve patients or the public in the design, or conduct, or reporting, or dissemination of plans of our research. The Cardiff and Vale Local Health Board Audit department approved the collection and use of this data for analysis without explicit consent from patients.

## Data Availability

Raw data is available upon request to the authors in reasonable circumstances. Our raw data is located within Cardiff and Vale IT services.
